# Cooling rate affects supercooling points of overwintering spotted lanternfly eggs

**DOI:** 10.3389/finsc.2026.1916560

**Published:** 2026-08-13

**Authors:** Clarissa Dahl, Angie Ambourn, Robert C. Venette

**Affiliations:** 1 Department of Entomology, University of Minnesota, Saint Paul, MN, United States; 2 Minnesota Department of Agriculture, Saint Paul, MN, United States; 3 United States Department of Agriculture (USDA) Forest Service, Northern Research Station, Saint Paul, MN, United States

**Keywords:** cold tolerance, freeze avoidance, invasive species, non-native insects, overwintering

## Abstract

Spotted lanternfly, *Lycorma delicatula* (White) is a planthopper (Hemiptera: Fulgoridae) that is native to China and invasive elsewhere in Asia and North America. The insect feeds on several plants and is an emerging pest of apples, grapes, walnuts, and other hardwoods. Forecasts of the ultimate geographic distribution for *L. delicatula* in Asia and North America, particularly its northern latitudinal limits, are uncertain because of limited information about the cold tolerance of this insect. Cooling rate can affect supercooling point, the temperature at which bodily fluids begin to freeze and a common reference point for insect cold tolerance studies. This study measures changes in supercooling point of overwintering *L. delicatula* eggs in response to different cooling rates. Overwintering egg masses were collected from two field locations in Virginia, USA in November and December 2024. Supercooling points of individual eggs were measured with contact thermocouple thermometry at cooling rates of 0.033, 0.5, and 1 °C/min. The distribution of supercooling points changed with different cooling rates and were negatively related to the cooling rate. At a cooling rate of 0.033 °C/min, SCPs ranged from -28.4 to -22.9 °C and, at 1 °C/min, from -29.2 to -24.7 °C. If supercooling points reflect lethal temperatures for *L. delicatula*, relatively rapid cooling may slightly overestimate the cold tolerance of the insect and inflate the estimated geographic area where overwintering might be successful. Such risk-averse forecasts may be useful for biosecurity efforts.

## 1 Introduction

Supercooling point (SCP) remains a core metric in the assessment of insect cold-tolerance strategies and, occasionally, of insect cold hardiness (1, 2). SCP is the lowest temperature an insect will reach before the spontaneous freezing of bodily fluids begins and the latent heat of crystallization is released. SCP is routinely measured through a combination of contact-thermocouple thermometry and temperature ramping, in which insects are gradually cooled until an exotherm, or sudden rise in temperature, is detected (2). Several factors can influence the SCP in insects including the presence of ice-nucleating agents, the extent of dehydration, the production of cryoprotectants, a recent history of cold exposure, and the rate of cooling (3–7).

Because the onset of freezing depends on time and temperature, Salt (8) recommended a standard cooling rate of 1 °C/min to facilitate comparisons among studies that measure SCPs of insects because he considered the rate to be “suitable and convenient.” He demonstrated that the cooling rate systematically affected the expected SCP for the wheat stem sawfly, *Cephus cinctus*, such that a doubling of the cooling rate lowered the mean SCP by 0.24 °C (8). A cooling rate of 1 °C/min remains common in studies of insect cold tolerance, but it has been criticized for its lack of ecological relevance given the rarity with which such cooling rates are experienced in nature (2, 9, 10). Thus, cooling rates between 0.1 and 0.5 °C/min have been recommended (2), but a standard alternative to the 1 °C/min cooling rate has not been agreed upon.

Salt (8) acknowledged that the use of cooling rates in addition to 1 °C/min may be appropriate in studies of insect cold hardiness. In theory, within limits, the onset of freezing should occur at lower temperatures if the cooling rate is increased (11), but empirical studies have reported inconsistent effects of cooling rate on SCP. For example, the expected SCP of fourth-instar *Trogoderma graminarium* declined as did trehalose concentration as cooling rate increased (12). Similarly, pupae and adults, but not larvae, of *Plodia interpunctella* had a 1 °C reduction in SCP when cooling rate was increased from 0.3 to 1.0 °C/min (13). *Alaskozetes antarcticus* and *Cryptopygus antarcticus* had bimodally-distributed SCPs; the expected SCPs declined as cooling rates increased for the “low groups”, i.e., individuals with SCPs in the lower peak, but not for the “high groups” (3). Worland (14) found a more complex response of SCP to cooling rate for *Tullbergia antarctica*; SCP was greater when cooled at 0.1 °C/min than at 0.025 °C/min but decreased as cooling rate increased from 0.1 °C/min to 8 °C/min. Other studies have directly compared different cooling rates and have shown little or no effect on SCP (15–19).

Taken alone, SCP may have little ecological relevance, regardless of the rate at which insects were cooled, given the potentially complex responses of insect species to cold exposure or freezing (9, 20). For freeze-avoidant species, i.e., those species that are chill tolerant and freeze intolerant, SCP is ecologically relevant because it is a lethal temperature for an individual. However, freeze-tolerant insects survive the onset of freezing but may experience lethal temperatures below the SCP. Conversely, chill-susceptible species die before the onset of freezing (i.e., at temperatures greater than the SCP). Sømme (21) conjectured that most insect species are freeze-intolerant.

**Figure 1:**
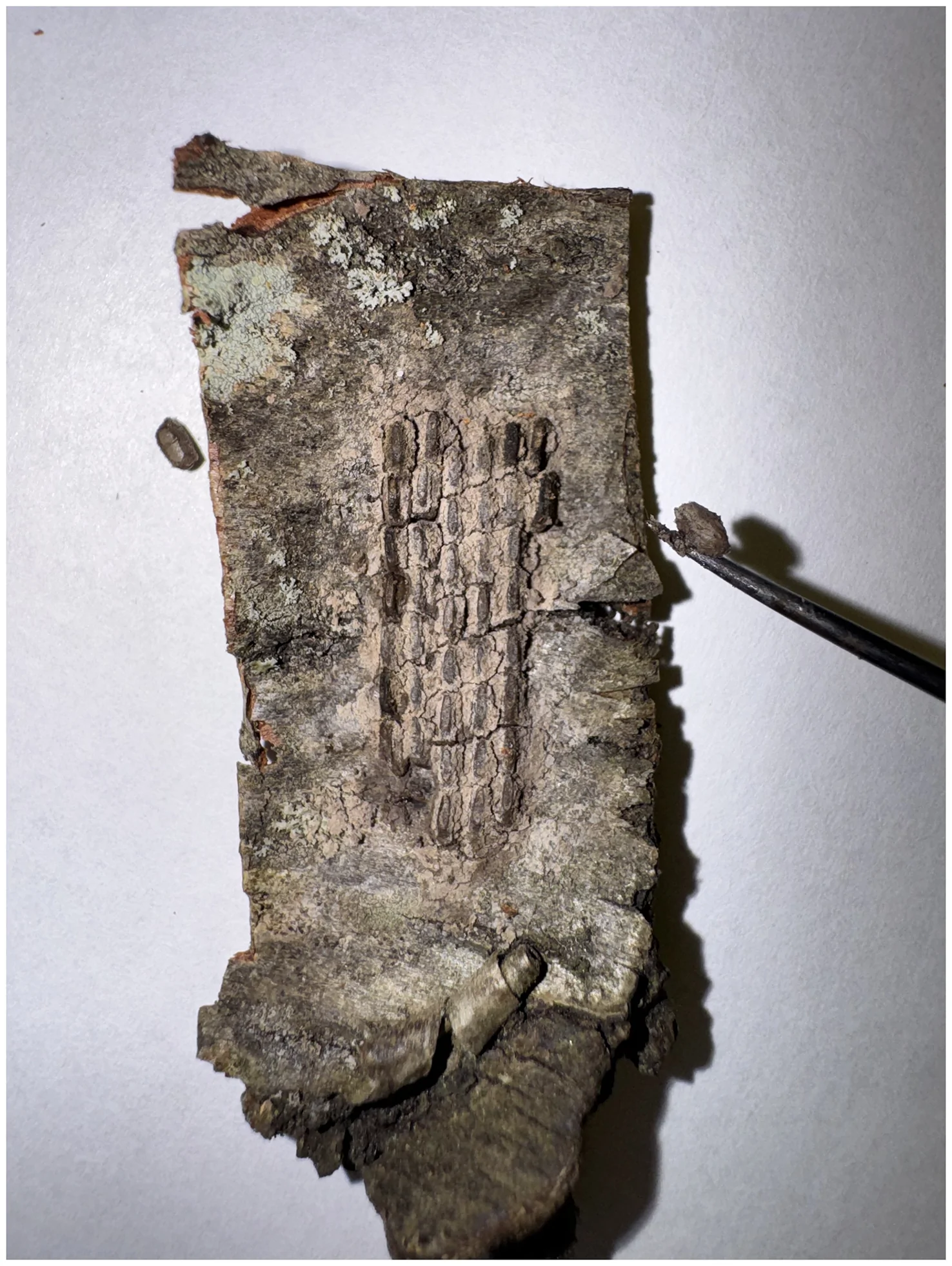
Egg removal from an overwintering egg mass of spotted lanternfly, *Lycorma delicatula*, on a cut bark surface.

Spotted lanternfly, *Lycorma delicatula* (White) [Hemiptera: Fulgoridae], is native to China and an emerging pest in South Korea, Japan and North America (22–25). The insect can affect economically important apple, *Malus domestica*; grape, *Vitis* spp; walnuts, *Juglans* spp; and other hardwood tree species (26). Abundance of this insect is closely related to the availability of tree of heaven, *Ailanthus altissima* (27). Projections of the ultimate geographic distribution of the insect in North America suggest that the climate of portions of New England and central North America may be unsuitable for this insect (5, 28, 29). Projections for northern latitudes and high elevations are constrained, in part, by limited detailed knowledge about the cold tolerance of this insect. *Lycorma delicatula* overwinter as diapausing eggs in masses covered by a layer of wax (30), often on the surface of a host plant (31) (**Figure 1**). At this stage, the insect is likely to be chill susceptible, though mortality may occur near the SCP (32). The lowest SCP reported for *L. delicatula* eggs was -27.7 °C when measured with a cooling rate of 0.5 °C/min (32). We assessed how cooling rate affects SCP of *L. delicatula* eggs and hypothesized that SCP would decrease as cooling rate increased. We tested this hypothesis with eggs collected in November and December 2024 from two populations of *L. delicatula*.

## 2 Methods

### 2.1 Insect collection

We sampled *L. delicatula* eggs from two sites: Roanoke, Virginia, USA (“south VA;” latitude, longitude: 37.2708018, -80.0198704) and Winchester, Virginia, USA (“north VA;” 39.213629, -78.151285). Roanoke is in USDA Plant Hardiness Zone 7b at an elevation of 216 m above sea level. In winter (December – February) of 2024/2025, air temperatures averaged 2.18 °C and ranged from -5.56 to 8.33 °C per the Roanoke-Blacksburg Regional Airport weather station (KROA), about 7 km northeast of our sampling site. Winchester is in Plant Hardiness Zone 7a in the Shenandoah Valley at an elevation of 298 m. Air temperatures averaged 0.61 °C, ranging from -9.44 to 6.11 °C in winter of 2024/2025, per the Winchester Regional Airport weather station (KOKV), about 6 km south of the sampling site.

Egg masses were collected from south VA on 19 Nov and 16 Dec 2024 and from north VA on 25 Nov and 16 Dec 2024. Egg masses were collected arbitrarily from host trees (most commonly, tree of heaven; autumn olive, *Elaeagnus umbellata*; black cherry, *Prunus serotina*; Callery pear, *Pyrus calleryana*; hackberry, *Celtis occidentalis*; Osage orange, *Maclura pomifera*; and red maple, *Acer rubrum*). The section of tree bark on which eggs were laid was peeled or cut from the tree (**Figure 1**); cut sections were ~ 2.5 – 7.6 cm in length. About half of the egg masses were from the bark of dead trees. Egg masses on bark were wrapped in paper towels and/or plastic wrap, double bagged, placed in Styrofoam coolers with an ice pack, and priority shipped to a biosecurity level-2 laboratory in St. Paul, Minnesota, USA. Eggs were in transit ≤ 2 days. All shipping and handling followed conditions described in the US Department of Agriculture, Animal and Plant Health Inspection Service permit number 526-24-309-38644. Upon receipt, egg masses were unwrapped, placed in Petri dishes that were sealed with Parafilm, and stored in a dark 3 °C incubator until experiments began.

To remove individual eggs from egg masses, we used a paint brush to remove the waxy egg covering. We whittled the wooden end of cotton swab to a flat edge and used it to gently pry individual eggs off the bark. Insect mounting pins dipped in small amounts of high-vacuum grease were used to transfer individual eggs into Petri dishes. Eggs from different egg masses were kept separately. Eggs were typically picked immediately prior to testing, though some eggs were picked and stored in the dark at 3 °C for up to 4 days.

### 2.2 Supercooling point measurements

For each shipment, we used contact thermocouple thermometry to measure the SCPs of eggs at three cooling rates: 0.033, 0.5, and 1 °C/min. Cooling rates were selected based on previously published methods (8). Each egg was attached to the end of a 0.127 mm diameter (36 AWG) type-T copper-constantan thermocouple with a bit of high-vacuum grease. A thermocouple was threaded through a customized plastic dowel fit with an O-ring that allowed us to seal each egg and thermocouple inside a 1.5 mL microcentrifuge tube, as originally described in Stephens et al. (33). These units were then placed into 18 x 180 mm Kimex glass test tubes in a 6 x 4 holding rack inside a chiller bath (A40, Thermo Fisher Scientific, Waltham, MA) filled with silicone circulating bath liquid (sil-180, Thermo Fisher Scientific). After placing up to 24 eggs with thermocouples into the chiller bath, we cooled eggs from room temperature to 3 °C at 1 °C/min before switching to the designated cooling rate. Runs at 0.033 °C/min required 1,000 minutes and occurred overnight. We verified that cooling rates stayed constant, although at the coldest temperatures (below about -28 °C) the cooling rate began to slow slightly. During runs, egg temperatures were recorded once per second with a multichannel data logger (USB-TC, Measurement Computing, Norton, MA), which had been calibrated and tested with InstaCal software (accuracy ±0.25 °C); data were logged and visualized by using Tracer-DAQ software (Measurement Computing, Norton, MA). SCPs were identified as the lowest temperature each egg reached before an exotherm occurred.

For each combination of site, month, and cooling rate, we conducted 2–3 supercooling runs within 16 days of collection, determining the SCP of 23–47 eggs from 3–6 egg masses, for a total of 407 eggs from 18 egg masses. Eggs from each mass were selected arbitrarily and arranged randomly in the chiller bath. A unique designator for each egg mass was assigned and recorded so that we could account for maternal effects in statistical models. The chiller bath malfunctioned in three runs of the slowest cooling rate before all eggs had supercooled: one run for north VA from December 2024 and both runs for south VA from December 2024. No data (46 observations) from these runs were analyzed.

### 2.3 Statistical analysis

Analyses were conducted in *R* v4.3.2 (34), and the “tidyverse” suite of packages was used for all data manipulation and visualization (35). Some SCPs were unusually warm, and preliminary testing suggested that such eggs may have been damaged during processing. SCPs of eggs that were intentionally pierced with an insect mounting pin ranged from -18.2 to -10.2 °C (n=4), values ≥8 °C than the mean SCP of other eggs measured at the same time. Because physical damage to the egg was not always evident and known physical damage to the egg elevated the SCP, we excluded observations that were more than 1.5-times the interquartile range above the third quartile (22 cases) for SCPs measured from the same site and month of collection from further analysis, as per Tukey (36). A total of 385 observations remained.

Descriptive statistics for the remaining data were calculated by using the *R* package “e1071” (37). We used the R package “boot” to perform bootstrapping of the data to test effects of cooling rate on differences in properties of SCP statistical distributions: mean, median, variance, skewness, and kurtosis. We used 10,000 iterations with replacement and downsampling (88 draws per iteration) to account for uneven sample sizes. Properties were considered significantly different (α=0.05) when 95% confidence intervals around paired differences did not include 0.

A preliminary Levene’s test for homogeneity of variance from the *R* package “car” (38) revealed differences in the variance of SCPs at different cooling rates (F = 3.79, P = 0.02, df=2; **Table 1**), so we applied a Box-Cox transformation to SCP (y), where y′ = ([-*y*]*^λ^*-1)/*λ*, and *λ* = 3. After transformation, no statistical differences in variance were detected (F = 2.82, P = 0.06, d.f. = 2). So, we used the R package “lme4” (39) to fit a linear mixed model in a stepwise approach and describe the effect of cooling rate on expected SCP. Candidate independent variables were *log_2_*(cooling rate), as per (8), site, month and all two-way and three-way interactions. An identifier for egg mass was treated as a random effect.

**Table 1:** Effect of cooling rate on properties of the statistical distribution of supercooling points of overwintering *Lycorma delicatula* eggs.

Property	Cooling rate (°C/min)		
	0.033	0.5	1.0
Mean	-26.6a	-27.0b	-27.2b
Median	-26.8a	-27.1ab	-27.1b
Variance	1.37a	1.03ab	0.76b
Skewness	0.99a	0.54ab	0.23b
Kurtosis	0.95a	0.10a	0.19a
n	88	140	157

Rows followed by the same letter are not significantly different (α=0.05) based on comparisons from 10,000 bootstrap simulations.

## 3 Results

**Figure 2:**
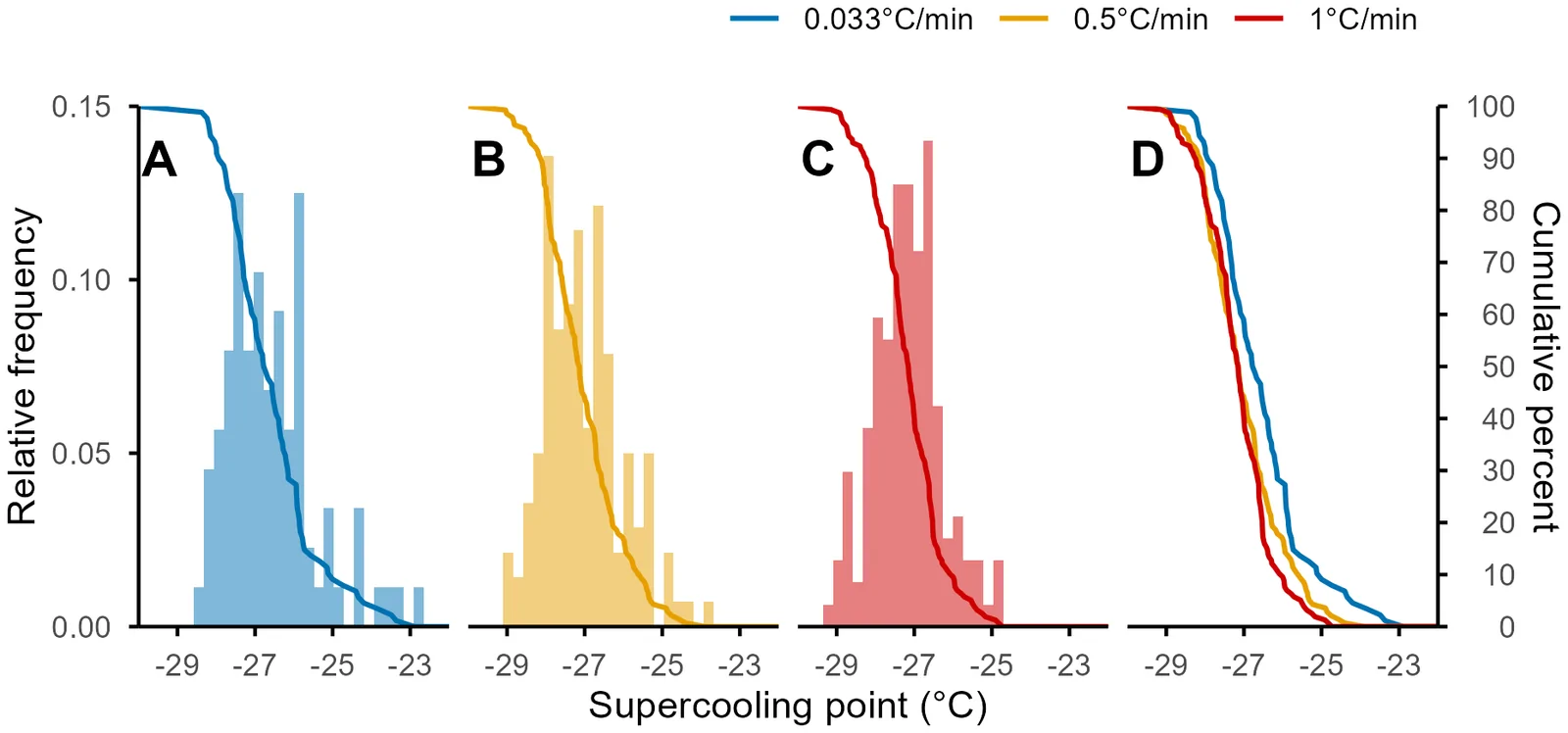
Relative frequency (bars) and cumulative percentage (lines) of supercooling points for overwintering *Lycorma delicatula* eggs cooled at **(A)** 0.033 °C/min; **(B)** 0.5 °C/min; and **(C)** 1 °C/min. Panel **(D)** overlays cumulative percentages of supercooling points from different cooling rates to facilitate comparisons among the tails of the distributions.

Cooling rate affected the SCP of overwintering *L. delicatula* eggs. At a cooling rate of 0.033 °C/min, SCPs ranged from -28.4 to -22.9 °C; at 0.5 °C/min, from -29.0 to -23.9 °C; and at 1 °C/min, from -29.2 to -24.7 °C (**Figure 2**). The mean, median, variance, skewness, and kurtosis, parameters that characterize the shape of the distribution, generally were greater for eggs cooled at 0.033 °C/min than at 1.0 °C/min, with parameters for eggs cooled at 0.5 °C generally being intermediate (**Table 1**). For the fastest cooling rate relative to the slowest, the mean SCP was 0.6 °C less; the median SCP was 0.3 °C less; and variance was approximately half (**Table 1**). Skewness, a measure of the symmetry of the distribution, was nearly five-fold less (**Table 1**). At the slowest cooling rate SCP has a stronger right skew (**Figure 1**; **Table 1**), while at the most rapid cooling rate, SCPs were distributed more symmetrically about the mean (**Figure 2**). Excess kurtosis, a measure of the weight of the tails of the distribution, was not statistically different at any cooling rate but was numerically greatest at the slowest cooling rate and least at the intermediate cooling rate (**Table 1**; **Figure 2**). This value describes the likelihood of observing values far from the mean if additional samples were to be collected.

**Figure 3:**
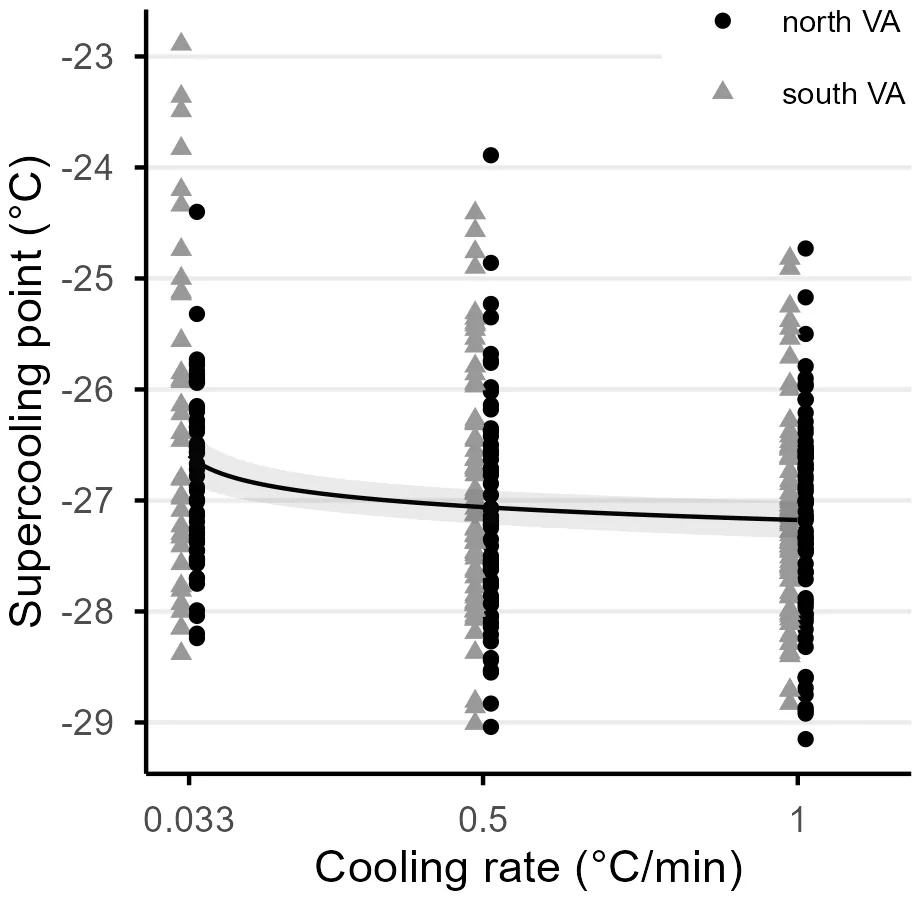
Relationship between supercooling point for overwintering eggs of *Lycorma delicatula* from north and south Virginia (VA), USA and cooling rate. Observations from November and December 2024 are combined and jittered about the cooling rate for visualization. The expected line (± 95% confidence interval) is back transformed.

The expected SCP of *L. delicatula* eggs decreased as cooling rate increased in a curvilinear pattern (**Figure 3**). We did not detect an effect of month, site, or any of their interactions on the expected SCP (Z ≤ 1.332; d.f. ≥ 141; P≥0.23); these were removed from the final model. The relationship followed the expression: *y*′=*b_0_* + *b_1_x*, where *b_0_* = 6,689.81 ± 62.62 (SE), *b_1_* = 85.31 ± 18.76, and *x* is *log_2_*(cooling rate). Both intercept and slope differed from 0 (*b_0_*: Z = 106.8, P<0.001; *b_1_*: Z = 18.8, P<0.001). Cooling rate accounted for 5.0% of the total variation in SCP; the identity of the egg mass explained an additional 2.1%. When back-transformed to remove the adjustment on *y*, the equation became y=-6.35 (78.42+*x*)^1/3^ and is shown in **Figure 3** with SCPs partitioned by site to illustrate the similarity in response across both collection locations.

## 4 Discussion

Our findings are in broad agreement with Salt (6). Specifically, we reaffirmed that cooling rate can affect the expected SCP of overwintering insects. Our results suggest a subtle non-linear response of the SCP of overwintering *L. delicatula* eggs to cooling rate (**Figure 3**). Even so, a doubling of the cooling rate lowered the expected SCP by ~0.12 °C within the range of our treatments. This pattern comports with theory. As liquids are chilled, they lose kinetic energy, and the probability that molecules of pure water will spontaneously form a crystallization nucleus, a process known as homogenous nucleation, increases with time below critical temperatures (40). So, with a slower cooling rate, the time at critical temperatures increases, and freezing can begin at greater temperatures (41). For insects, heterogeneous nucleation, with foreign substrates facilitating the formation of the crystallization nucleus, is more likely (40). Rapid cooling can interfere with thermal equilibrium within an insect and mask the effects of nucleators, delaying the onset of freezing to lower temperatures.

Cooling rate explained a relatively small amount (5%) of the variation in SCP for *L. delicatula* eggs in our study. We concur with the suggestions of Salt (6) that a cooling rate of 1.0 °C/min is efficient for measuring SCPs and that exploration of other cooling rates may be worthwhile, depending on the nature of the question of interest. In our case, the exploration of the effect of cooling rate on SCP is preparatory work for the measurement of other cold tolerance metrics for this insect. Understanding the relationship between cooling rate and the distribution of SCP allows for comparison of SCP of *L. delicatula* measured at different cooling rates, as recommended by Salt (8).

Our results suggest that the onset of freezing of overwintering *L. delicatula* eggs occurs at slightly lower temperatures than previously reported. Although the mean SCPs we recorded, regardless of cooling rate, were comparable to the mean of -25.5 °C (± 2 °C) reported by Turbelin et al. (32), the median values we found were approximately 2 °C lower than the previously reported median of -24.4 °C. Further, our lowest SCPs were -29.2 to -28.4 °C, depending on cooling rate, all less than -27.7 °C, the previously reported low SCP for overwintering *L. delicatula* eggs when cooled at 0.5 °C/min (32).

Overwintering *L. delicatula* eggs are likely to be chill susceptible, but with mortality occurring just before or during the onset of freezing (32). So, the distribution of SCPs could directly affect the proportion of the population that would be expected to survive brief exposure to a particular sub-zero temperature. With a slower cooling rate, a right-skewed distribution of SCPs became evident, and freezing began at greater temperatures than when individuals were cooled at 1.0 °C/min (**Table 1**, **Figure 2**). For example, when cooled at 0.033 °C, 9% of individuals started to freeze upon exposure to -25.0 °C, but only 4% or 1% started to freeze when cooled at 0.5 °C/min or 1 °C/min (**Figure 2**). In contrast, 95% of individuals had started to freeze at -28.1 °C when cooled at 0.033 °C/min. But, when *L. delicatula* eggs were cooled at 0.5 or 1.0 °C/min, 95% of eggs had started to freeze at -28.4 or -28.7 °C, respectively (**Figure 2**). These differences are less than the variation in temperatures among microsites that might be expected at a location (42) and may be of limited ecological relevance.

Cold tolerance information has long been of interest to help explain the distribution and abundance of insects [e.g., (43)]. Timely cold tolerance assessments are important for invasive insect species, especially those that may not have reached the limits of their geographic distribution (44, 45). This information can inform process-oriented ecological niche models to forecast where an invasive pest might encounter climatically suitable habitat for establishment. Such information is useful for biosecurity efforts to determine, for example, where quarantines might be appropriate or where to conduct surveillance. Estimates of cold tolerance can prevent errors of omission, i.e., forecasting an area to be unsuitable for overwintering when it is suitable. If supercooling points reflect lethal temperatures for *L. delicatula*, relatively rapid cooling may slightly overestimate the cold tolerance of the insect and inflate the estimated geographic area where overwintering might be successful. Such risk-averse forecasts may be useful for biosecurity efforts.

In North America, *L. delicatula* continues to spread and establish from its point of first detection in Berks County, Pennsylvania, USA, with infested counties generally remaining south of 43° N latitude (46). This insect has moved and established as far west as Cook County, IL as of July 2025 (46). Although *L. delicatula* has been intercepted in southcentral and southeastern Canada (32), as of October 2025, it was not yet considered established there (47). If SCP is indicative of winter survival potential, we expect additional northward spread of the insect in North America. Future work on the relationship(s) between mortality, temperature, and time is needed to provide confidence in that expectation.

In conclusion, cooling rate affected SCP of overwintering eggs from *L. delicatula.* SCPs were generally greater and exhibited greater variation when measured with lower cooling rates. However, temperatures needed to cause 95% of individuals to begin to freeze varied by 0.6 °C or less among the cooling rates we explored. Thus, the standard 1 °C cooling rate is efficient for SCP measurements for this insect. Other cooling rates may be needed to accurately forecast overwintering mortality of eggs.

## Data Availability

The raw data supporting the conclusions of this article will be made available by the authors, without undue reservation.
